# Lingual Raynaud’s Phenomenon: A Case Series and Literature Review

**DOI:** 10.3390/jcm15051738

**Published:** 2026-02-25

**Authors:** Marie Morard, Nicolas Brebion, Marc Lambert, Elisabeth Diot, Bertrand Lioger, Jean-Manuel Kubina, Christian Agard, Olivier Espitia

**Affiliations:** 1 CHU Nantes Department of Internal and Vascular Medicine, CNRS UMR 6291, INSERM UMR 1087, L’Institut du Thorax Team III Vascular and Pulmonary Diseases, Nantes Université, F-44000 Nantes, France; 2 Department of Vascular Medicine, CHCL Cote de Lumière, F-85110 Les Sables d’Olonne, France; 3 CHU Lille, Département de Médecine Interne et Immunologie Clinique, Centre de Référence des Maladies Auto-Immunes Systémiques Rares du Nord et Nord-Ouest, Méditerranée et Guadeloupe (CeRAINOM), University LILLE, F-59000 Lille, France; 4 Department of Internal Medicine, CHRU de Tours, F-37000 Tours, France; 5 Department of Internal Medicine, CH Blois, F-41000 Blois, France; 6 Department of Vascular Medicine, CHD Vendée La Roche sur Yon, F-85000 La Roche sur Yon, France; 7 CHU Nantes Department of Internal and Vascular Medicine, Nantes Université, F-44000 Nantes, France; christian.agard@chu-nantes.fr

**Keywords:** Raynaud’s phenomenon, tongue, lingual, systemic sclerosis, radiotherapy, idiopathic, dysarthria, vasodilator treatment

## Abstract

**Background**: Raynaud’s phenomenon of the tongue is a rare manifestation that may be associated with systemic diseases. The clinical manifestations, etiologies and management of this condition are poorly described. **Methods**: We report 10 cases of lingual Raynaud’s phenomenon (LRP) and 26 cases from a structured literature review. **Results**: In 38.8% of cases, the LRP occurred in the context of a previously diagnosed systemic sclerosis; 16.6% followed radiotherapy for head and neck cancer; and 27.8% of patients presented with an idiopathic-like form. The manifestations classically included a syncopal phase (91.7%) associated with hypoesthesia (88.9%) and possible dysarthria (52.8%). Atypical presentations with a primary cyanotic phase were also observed, particularly in the context of vasculitis, notably cryoglobulinemic vasculitis (four patients). Active smoking was a significant triggering factor in idiopathic forms (60%). Across all patients—both primary and secondary forms—the most common triggering factor was cold exposure (75%). Vasodilator use showed good efficacy and should be considered for all highly symptomatic patients. **Conclusions**: In summary, LRP is more frequently associated with systemic sclerosis, manifesting as blanching of the tongue associated with hypoesthesia and dysarthria in more than half of cases. Vasodilators may reduce symptoms. Larger studies are needed to confirm these findings.

## 1. Introduction

Raynaud’s phenomenon (RP) is a manifestation linked to changes in microcirculation with a vasospasm that occurs in the context of very distal arteriopathy. Raynaud’s phenomenon mostly affects the fingers. It is usually idiopathic but can reveal autoimmune diseases, such as scleroderma or mixed connective tissue disorders, and is found in multiple connective tissue disorders. It is a classic, if not systematic, feature of systemic sclerosis [1,2], with the presence of positive antinuclear antibodies, most often with anti-centromere or anti-SCL 70 specificity. Other well-known causes of RP include toxic (e.g., smoking) or iatrogenic (e.g., beta-blockers) factors, and it can also occur following trauma of the digital arteries or palmar arches. It is typically described in the fingers and sometimes the toes, but more atypical locations such as the tongue have also been reported. Raynaud’s phenomenon of the tongue remains a very rare manifestation, with only a few cases described in the literature.

The aim of this study was to report a case series of lingual RP (LRP) associated with cases from the literature identified through a structured review in order to describe the clinical presentations and etiologies and to report the treatments used.

## 2. Patient and Method

### 2.1. Patients

**Figure 1:**
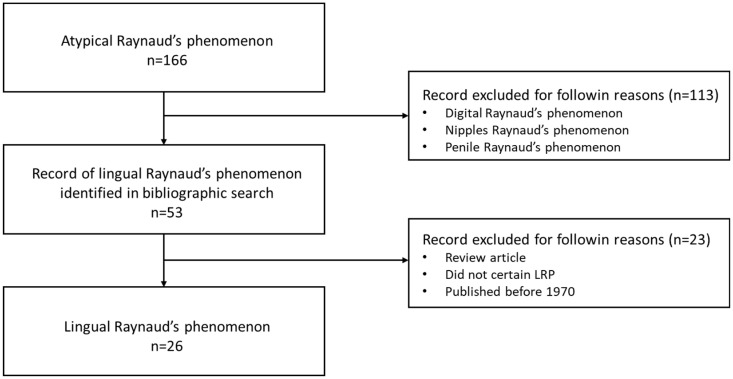
Patient selection flowchart from the literature with lingual Raynaud’s phenomenon.

This multicenter retrospective study included patients presenting with Raynaud’s phenomenon of the tongue. Members of the French Society of Vascular Medicine referred the cases based on the following inclusion criteria: the presence of a paroxysmal color change of the tongue toward pallor and/or cyanosis, with a spontaneously reversible nature, confirmed by a physician of a referral center experienced in managing patients with Raynaud’s phenomenon of the extremities (Figure 1).

This study was conducted in compliance with the Declaration of Helsinki principles and received ethics approval from the local ethics committee of the University Hospital of Nantes (20170609). Each patient included in this study received written information, and no patient objected to this study. The need for written informed consent was waived by the ethics committee because of the retrospective study design (French public health code article: L 1121-1).

### 2.2. Literature Review

We conducted a literature review, initially identifying references by searching the PubMed database using the keywords “atypical Raynaud’s phenomenon”, “tongue Raynaud’s phenomenon”, and “lingual Raynaud’s phenomenon”. The inclusion criteria were the presence of Raynaud’s phenomenon of the tongue, defined by a paroxysmal change in the color of the tongue. To be included in this study, cases of Raynaud’s phenomenon of the tongue had to be sufficiently documented with investigations to determine a secondary etiology specified in the report. Cases of atypical Raynaud’s phenomenon not affecting the tongue were excluded, such as cases of patients with Raynaud’s phenomenon in the fingers with tongue symptoms but no change in tongue color. Out of 166 references, we selected only 53 articles that specifically addressed Raynaud’s phenomenon of the tongue, after excluding other atypical forms of Raynaud’s affecting the fingers, nipples, or penis. Among these 53 articles, we further included only original articles—excluding literature reviews—published after 1970, in which the diagnosis of Raynaud’s phenomenon of the tongue was confirmed.

### 2.3. Statistical Analysis

We compiled the results to report the clinical and laboratory manifestations of LRP in our cases and in the literature cases.

## 3. Results

### 3.1. Present Report

This study included 10 cases of LRP from the vascular medicine cohort and 26 cases from the literature. Of the 10 cases of LRP from our cohort, 5 cases were associated with systemic sclerosis, 1 case with giant cell arteritis, 1 case with head and neck cancer treated with radiotherapy, and 3 idiopathic forms (Table 1).

In our cohort, all patients underwent at least testing for antinuclear antibodies (except for case 9, who had a normal capillaroscopy but no documented antibody testing). Idiopathic cases had capillaroscopies considered ‘normal’ and negative antinuclear antibodies. No Doppler ultrasound assessments were performed.

Case 6: We report the case of a 68-year-old female patient at the time of initial manifestations of LRP. She was a former smoker, with an estimated cumulative tobacco exposure of 40 pack-years, and reported no other toxic exposures. She had experienced a relative weight loss of 6 kg over the same period. Since the age of 38, she had a primary-appearing Raynaud’s phenomenon affecting the fingers. This new clinical manifestation occurred in the context of a broader symptomatology highly suggestive of giant cell arteritis, including occipital and temporal headaches, general health deterioration, biological inflammatory syndrome, and scalp necrosis. The cephalic presentation was associated with florid vasculitis involving the aorta, the supra-aortic trunks, the axillo-subclavian arteries—complicated by a humoral thrombosis—and the superficial femoral arteries. All of her symptoms, including Raynaud’s phenomenon of the tongue, improved with corticosteroid therapy, without any need for vasodilators.

**Figure 2:**
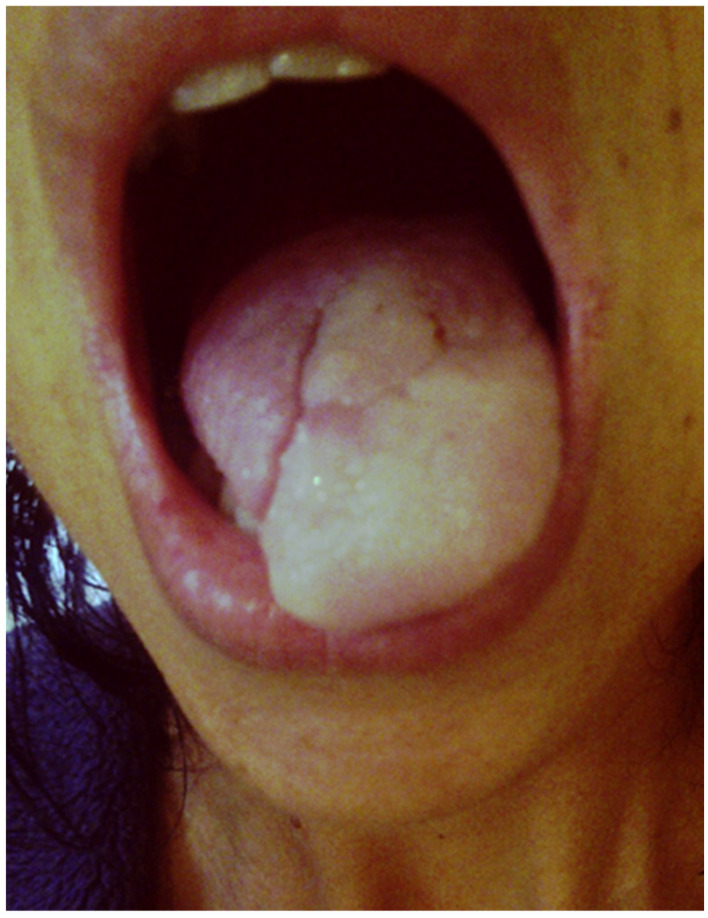
Raynaud’s phenomenon localized to one hemilingual region: clinical manifestation.

Case 7: We report the case of a 38-year-old female patient at the onset of Raynaud’s phenomenon of the tongue, who had undergone surgery and radiotherapy for tongue cancer one year earlier (Figure 2). There was no evidence of a connective tissue disease, with negative antinuclear antibodies, although she had developed Raynaud’s phenomenon of the fingers at the age of 37, around the time she had lost approximately 12 kg at cancer diagnosis. She was a former smoker, with an estimated cumulative exposure of 10 pack-years, and reported no other toxic exposures. Her presentation of Raynaud’s phenomenon of the tongue consisted solely of a syncopal phase, associated with hypoesthesia and dysarthria, lasting up to one minute. Episodes could occur spontaneously but were often triggered by exposure to cold. She did not undergo a trial of vasodilator therapy. It should be noted that the color change involved only the left part of the tongue, on the same side as the area treated with radiotherapy.

Cases 8–10: We report three cases of idiopathic forms of Raynaud’s phenomenon of the tongue, including two men aged 54 (case 8) and 48 (case 9) at first presentation and one woman aged 48 (case 10). All were active smokers, with case 9 also reporting cannabis use. There were no finger manifestations in case 9, while cases 8 and 10 had a longstanding primary-appearing Raynaud’s phenomenon of the fingers. The syncopal phase was consistently present with hypoesthesia and dysarthria; cases 8 and 10 also exhibited a cyanotic phase, although attacks never lasted more than 10 min. None had a history of neoplastic disease or evidence of connective tissue disease, with negative antinuclear antibody testing and negative capillaroscopy. Attacks were triggered by cold exposure, with possible spontaneous episodes reported in case 8.

### 3.2. Review of Literature Associated with the Case Series

The 26 cases documented in the literature were combined with our 10 previously reported cases for the analysis of characteristics and phenotypes in 36 patients [3,4,5,6,7,8,9,10,11,12,13,14,15,16,17,18,19,20,21,22,23,24,25,26].

We identified nine cases of systemic sclerosis, five cases related to cervicofacial radiotherapy, and seven cases classified as idiopathic. There was one exceptional case of DRESS [3] syndrome and four cases of connective tissue diseases other than systemic sclerosis [6,7,16,25]. These presented atypically, with an initial cyanotic phase and positive cryoglobulins, along with associated markers of activity (low complement levels and elevated rheumatoid factor), suggesting forms of Raynaud’s phenomenon of the tongue secondary to cryoglobulinemic vasculitis.

By combining the cases from the literature with our series, we were able to assess patient phenotypes for the most represented forms, with 14 cases of systemic sclerosis (5 from our series and 9 reported in the literature), 6 cases of ENT cancer (1 from our series, Case 7, and 5 reported in the literature), and 10 idiopathic forms (3 from our series and 7 reported in the literature). These data are presented in Table 2.

Forms related to systemic sclerosis occur on average at the age of 36 years (median 32), consistently in patients with a prior history of Raynaud’s phenomenon affecting the fingers, which began on average at age 23 (median 23.5). The condition predominantly affects women, with a sex ratio of 1 male to 13 females. In our cohort, the cyanotic phase was consistently present compared to the radiotherapy and idiopathic groups. Spontaneous episodes were reported by nine patients (64.3%).

In the post-radiation and idiopathic forms, the underlying Raynaud’s phenomenon of the fingers seems to be less common compared to the scleroderma group.

Forms not related to connective tissue disease or radiotherapy-induced seem to be more associated with active smoking, although there are patients in whom long-term smoking cessation is achieved, which does not support a diagnosis consistent with thromboangiitis obliterans profiles. The three groups are comparable regarding former smoking and cumulative consumption estimated in pack-years.

Cold exposure is a major triggering factor (75% of patients), but the intake of cold food or drinks is not a provoking factor. Approximately half of the patients (47%) will experience spontaneous episodes, although these individuals often report an emotional trigger.

There is little use of vasodilators outside of the forms related to connective tissue diseases. A case has been reported in the literature of an attempt to use botulinum toxin in a patient presenting with Raynaud’s phenomenon of the tongue and chin, associated with torticollis following mandibulectomy and reconstruction for head and neck carcinoma, with adjuvant radiotherapy [5].

It should be noted that in post-radiation forms, vasospasm may affect only half of the tongue—or even a single quadrant of the tongue—on the same side as the area treated with radiation.

## 4. Discussion

This study reviewed all documented LRP cases in the literature, along with the analysis of 10 additional cases, making it, to our knowledge, the largest case series of Raynaud’s phenomenon of the tongue. We have presented the most frequent etiologies—systemic sclerosis and ear, nose, and throat (ENT) cancer treated by radiotherapy—as well as the background and phenotypic presentations of patients according to the etiology. The syncopal phase associated with hypoesthesia of the tongue and dysarthria is classic, but in the absence of an examination of the tongue during paroxysmal episodes of dysarthria, the diagnosis may be missed, leading to neurological investigations that ultimately yield negative results.

As with Raynaud’s phenomenon of the fingers, the association with tobacco use is common in primary (idiopathic) forms, and the phenomenon is frequently triggered by cold (27/36, 75%) but can still occur spontaneously (17/36, 47%). Vasospastic manifestations labeled as Raynaud’s phenomenon share the common feature of affecting acral locations (fingers, toes, tongue, nose, nipples, etc.). They result from overall cooling that induces vasospasm primarily in these extremities. There does not appear to be a link with a specific region or transient local exposure to a cold stimulus; the vasospasm remains a global and often generalized response (for example, Raynaud’s affecting both the tongue and fingers during the same episode). Therefore, cold food or beverages do not seem to trigger a vasospastic attack in the tongue.

Cyanotic phases occurring immediately (without a preceding syncopal phase) should primarily prompt the search for small- and large-vessel vasculitis, particularly cryoglobulinemic vasculitis or giant cell arteritis, depending on the rest of the clinical presentation.

The pathophysiology of post-radiation Raynaud’s phenomenon can be explained by the destructive effect of radiation on the endothelium, leading to a loss of modulation by chemoreceptors and resulting in vasospasm. It also involves radiation-induced destruction of the capillary bed, causing chronic tissue hypoxia and heightened sensitivity to vasospasm [21].

The use of vasodilators is limited outside of forms related to systemic sclerosis, but their effectiveness seems excellent in this group (78%), with a reduction in both the frequency and duration of events. Calcium channel inhibitor prescriptions, mainly nifedipine, could be extended to all patients depending on their level of discomfort, although the response to calcium channel inhibitors over time is difficult to establish using retrospective data in terms of the number and intensity of seizures.

The main limitation of our study is the potential publication bias, with the majority of reports published in rheumatology, internal medicine, or dermatology journals, and few oncology patients. Moreover, rare manifestations associated with systemic diseases are far more likely to be published than isolated idiopathic cases. Therefore, the apparent predominance of forms related to systemic sclerosis should be interpreted with caution due to this publication bias and does not seem to reflect the true epidemiology.

Our sample size remains limited, although, to our knowledge, this is the largest reported case series.

Another limitation to note is the lack of consistency in the investigations of tongue Raynaud’s phenomenon among the different practitioners in our cohort. At a minimum, testing for antinuclear antibodies and performing capillaroscopy was almost systematic, except in cases with an obvious history of head and neck radiotherapy. However, some patients underwent more extensive biological investigations, including scleroderma DOT testing, cryoglobulin screening, and complement consumption assessment. In the absence of a comprehensive autoimmune work-up, the evaluation of patients could be considered incomplete, and therefore, classifying the Raynaud’s phenomenon as “idiopathic” may be incorrect.

The aim of this study is not to compare the different forms of Raynaud’s phenomenon of the tongue—whether associated with systemic sclerosis, post-radiation, or idiopathic—but rather to identify clinical presentations suggestive of each form in order to guide the referring physician toward a diagnosis. Nevertheless, it is agreed that all patients should undergo at least a capillaroscopy and testing for antinuclear antibodies before concluding that a case is an idiopathic form.

## 5. Conclusions

Raynaud’s phenomenon of the tongue has a relatively consistent presentation, typically involving a blanching (syncopal) phase associated with hypoesthesia and, in some cases, transient dysarthria. This phase resolves spontaneously. The cyanotic phase may appear initially or follow the syncopal phase and can be painful, presenting as dysesthesia.

The identification of Raynaud’s phenomenon of the tongue should prompt a search for an underlying connective tissue disease, particularly systemic sclerosis, especially in the presence of Raynaud’s phenomenon affecting the fingers. When the condition is isolated to the tongue—and sometimes localized to a specific segment—a history of neck and facial radiotherapy should be investigated. In the absence of strong evidence for either of these two diagnoses, an idiopathic form may be considered, with active smoking appearing to be an aggravating factor. Any atypical presentation, especially without a syncopal phase, should raise suspicion for small- or large-vessel vasculitis; in such cases, the clinical picture typically includes additional symptoms beyond Raynaud’s phenomenon of the tongue. Vasodilator treatment with nifedipine may represent a potential approach for improving patients’ symptoms in addition to cold protection measures and smoking cessation. These data must be confirmed by prospective studies with a large sample size.

## Figures and Tables

**Table 1:** Characteristics of newly reported scleroderma patients in our multicenter series.

	Case 1	Case 2	Case 3	Case 4	Case 5
Age at diagnosis of SS (y-o)	23	47	44	22	NA
Antinuclear antibody specificity	anti-Scl-70	non-specific	anti-centromere	anti-centromere	anti-Scl-70
Presence of fingers RP	Yes	Yes	Yes	Yes	Yes
Age at diagnosis of RP (y-o)	23	27	24	20	NA
Other clinical manifestations of SS	Digital ulcer Interstitial lung disease	Digital ulcer	None	None	Digital ulcer Interstitial lung disease PHTN
Maintenance therapy for the connective tissue disease	Prednisone	None	None	None	Prednisone MMF
Age at diagnosis of LRP (y-o)	32	32	45	20	45
Sex	Female	Female	Female	Female	Male
BMI Kg/m^2^	22.8	23.7	36.7	NA	NA
Weight loss contemporaneous with the onset of LRP	None	None	None	None	None
Active smoking at diagnosis of LRP	None	None	Yes	None	None
Characteristics of LRP					
Presence of a syncopal phase	Yes	Yes	Yes	Yes	Yes
Presence of a cyanotic phase	Yes	Yes	Yes	Yes	Yes
Presence of a lingual ulceration	None	None	None	None	None
Maximum duration of attacks (in minutes)	NA	10	5	3	1
Presence of dysarthria	Yes	Yes	Yes	None	None
Triggered by general cold exposure	Yes	Yes	Yes	Yes	Yes
Triggered by intake of cold food or drink	None	None	None	None	None
Spontaneous events	Yes	None	None	Yes	Yes
Treatments					
Vasodilator treatment	Yes	Yes	Yes	Yes	Yes
Vasodilator molecule used	Nifedipine	Nifedipine	Nifedipine	Ilomedine	Amlodipine
Effect of vasodilator treatment on LRP (reducing number and duration of attacks)	Benefic	Benefic	Benefic	Benefic	No benefit

Abbreviations: SS: systemic sclerosis; BMI: body mass index; LRP: lingual Raynaud’s phenomenon; RP: Raynaud’s phenomenon; MMF: mycophenolate mofetil; PHTN: pulmonary hypertension.

**Table 2:** Patient characteristics according to etiology of LRP; data are presented as n (%), mean, and median.

	Systemic Sclerosis n = 14	ENT Cancer with Radiotherapy n = 6	Idiopathic n = 10
Presence of fingers RP	14 (100%)	3 (50%)	4 (40%)
Age at diagnosis of RP (y-o)	22.8; 23.5	35.7; 37	24.3; 22
Age at diagnosis of LRP (y-o)	35.9; 32	52.7; 50.5	40.8; 48
Male gender	1 (7.1%)	1 (16.7%)	4 (40%)
BMI Kg/m^2^	27.7; 23.7	-	20.1; 20.1
Weight loss contemporaneous with the onset of LRP	0 (0%)	1 (16.7%)	1 (10%)
Active smoking at diagnosis of LRP	1 (7.1%)	0 (0%)	6 (60%)
Former smoking	2 (14.3%)	3 (50%)	5 (50%)
Cumulative consumption (pack-years)	23.0; 25	15.0; 15	37.2; 25
Presence of a syncopal phase	7 (87.5%)	6 (100%)	9 (90%)
Presence of a cyanotic phase	14 (100%)	3 (50%)	3 (30%)
Presence of a lingual ulceration	0 (0%)	0 (0%)	0 (0%)
Maximum duration of attacks (in minutes)	9; 10	6; 4	6; 3
Presence of dysarthria	9 (64.3%)	2 (33.3%)	4 (40%)
Triggered by general cold exposure	9 (64.3%)	6 (100%)	8 (80%)
Triggered by intake of cold food or drink	0 (0%)	1 (16.7%)	0 (0%)
Spontaneous events	9 (64.3%)	3 (50%)	4 (40%)
Initiated vasodilator treatment	8 (57.1%)	1 (16.7%)	2 (20%)
Positive effect of vasodilator treatment on LRP	7/8 (87.5%)	1/1 (100%)	0/0 (0%)

Abbreviations: ENT: ear, nose, and throat; BMI: body mass index; LRP: lingual Raynaud’s phenomenon; RP: Raynaud’s phenomenon.

## Data Availability

All data is provided within the article.
